# Architecture, goals and challenges of the Swiss Information System for Antibiotics in Veterinary Medicine (IS ABV)

**DOI:** 10.1093/jacamr/dlaf199

**Published:** 2025-10-27

**Authors:** Guy-Alain Schnidrig, Heinzpeter Schwermer, Dagmar Heim, Anaïs Léger, Gertraud Schüpbach-Regula

**Affiliations:** Veterinary Public Health Institute, Vetsuisse, University of Bern, Liebefeld, Bern, Switzerland; Graduate School of Cellular and Biomedical Sciences, University of Bern, Hochschulstrasse 4, Bern, Switzerland; Department of Animal Health and Animal Welfare, Federal Food Safety and Veterinary Office (FSVO), Liebefeld, Bern, Switzerland; Department of Animal Health and Animal Welfare, Federal Food Safety and Veterinary Office (FSVO), Liebefeld, Bern, Switzerland; Department of Animal Health and Animal Welfare, Federal Food Safety and Veterinary Office (FSVO), Liebefeld, Bern, Switzerland; Veterinary Public Health Institute, Vetsuisse, University of Bern, Liebefeld, Bern, Switzerland

## Abstract

**Background and objectives:**

In-depth knowledge of antibiotic treatments in animals is essential to effectively combat antimicrobial resistance. Notably, veterinary antibiotic sales in Switzerland have dropped by >50% in the past decade. However, a further breakdown by species or detailed livestock classes such as dairy or fattening cattle has so far not been possible because products are often authorized for use in multiple species and sectors. In 2020, the Swiss national monitoring system for antibiotic use (IS ABV) was introduced, which has substantially improved the availability of detailed data on antibiotic use. The aim of this study is to provide a high-level overview of IS ABV, its technical implementation and the current state of the system, highlighting its strengths and current and past weaknesses.

**Methods:**

To achieve this, we extracted antibiotic use data from the IS ABV surveillance system and analysed prescription-level data from 2020 to 2023, applying seasonal adjustments using ARIMA time series modelling.

**Results:**

We identified a significant decreasing trend in the number of prescriptions for companion and farm animals. In addition, the approximated reporting errors have decreased over time, indicating improved accuracy in documenting antibiotic use by veterinarians.

**Conclusions:**

These advances highlight the system’s effectiveness in improving data accuracy and in monitoring antibiotic management improvements in Swiss veterinary practices. By providing more reliable, timely data, IS ABV enables better monitoring of prescribing patterns and supports evidence-based decision-making. This enables the authorities to design targeted interventions and policies aimed at reducing unnecessary antibiotic use, thereby contributing to the fight against antimicrobial resistance.

## Introduction

Antimicrobial resistance (AMR) has been recognized as a major global health threat.^1^ The development of AMR is linked to antibiotic use (AMU) in both human and veterinary medicine.^2^ Therefore, detailed knowledge of antibiotic treatments in animals and robust surveillance of usage and resistance are crucial to guide interventions that combat the spread of AMR.^3^

Since 2009, the Federal Food Safety and Veterinary Office (FSVO) of Switzerland has been collecting and analysing annual veterinary sales data from pharmaceutical companies in Switzerland.^4^ These data were also reported to the European Surveillance of Veterinary Antimicrobial Consumption project (ESVAC)^5^ and were included in the ARCH-Vet report, which was published annually.^6^ Sales of antibiotics have reduced by >50% in the past 10 years. In addition, the use of critical antibiotics (i.e. macrolides, fluoroquinolones, cephalosporines of third and fourth generation) has been reduced by two-thirds. With these sales data, an overview on the usage of different antimicrobial classes could be displayed, and trends analysed.

However, a further breakdown by species or detailed livestock classes such as dairy or fattening cattle is not possible, as products are often authorized for use in multiple species and sectors and this information is missing from pharmaceutical sales data. To combat AMR, the Swiss government developed the Swiss Antibiotic Resistance Strategy in 2016,^7^ which aims to control antibiotic resistance. A regulatory framework was adopted (Heilmittelgesetz, HMG,^8^ Informationssystem Antibiotika in der Veterinärmedizin, IS ABV^9^ & Tierarzneimittel Verordnung TAMV^10^) to develop a national system for data collection on AMU in animals.

The information system for antibiotics in veterinary medicine (IS ABV)^11^ was launched up in late 2019, requiring veterinarians in Switzerland to register all prescriptions and use of antibiotics for farm animals and companion animals. When Switzerland introduced IS ABV, several monitoring systems for AMU in livestock (mainly fattening) were already in place in Europe—some for a long time—but there were none for companion animals.^12^ The European legislation for the collection of data on the use of antibiotics requires the systematic collection of data for cattle, poultry and pigs by 2025 and for companion animals by 2029.^13^ At that point in 2019, a total of 38 active AMU monitoring systems were in operation at farm level in 16 countries, with varying degrees of legal obligation.^14^ There are three main methods of collecting data on AMU: at the farm level, via the veterinary practice or via the pharmacies or wholesalers supplying the practices. Furthermore, different units of measurement are used within these systems, including mass-, dose- and count-based.^14^

The goal of IS ABV was to establish a system whereby all veterinarians in Switzerland would be required to submit mass-, dose- and count-based data on AMU in companion and farm animals. With its launch in late 2019 the first annual report analysing 2020 data was published in 2021 and updated annually thereafter.^11^ These reports provide a more comprehensive and detailed analysis of antibiotic data than can be obtained from sales data from pharmaceutical companies. However, analysis of the vast amount of antibiotic use data revealed several problems with data quality and the complexity of the analyses. For this study, we focused on prescription and veterinary practice data to provide a high-level overview of the IS ABV surveillance system and its overall performance.

This article presents an overview of the technical implementation of the IS ABV system, with a particular focus on its design, features, data quality, challenges and potential improvements since its inception in 2020. It begins by detailing the system’s reporting procedures and architecture, followed by an examination of the master data, which includes core standardized information such as approved drugs, veterinary practices and animal categories, as well as details of its structure, purpose and maintenance processes. The article also examines the annual prescribing patterns of Swiss veterinarians over the first four years, including the factors that may contribute to implausible prescribing. The aim of this article is to present the current state of the IS ABV system, highlighting its strengths and current and past weaknesses, in order to provide insights that can inform and improve the development of future AMU monitoring systems.

## Methods

### Analyses and data extraction

Analyses presented in this study were conducted using R (version 4.2.2) with R Studio (version 2023.09.0)^15^ Analyses are based on IS ABV data and sales data from pharmaceutical companies extracted on 1 August 2024 and cover the years 2020–2023. The data were cleaned and analysed only at prescription level (e.g. number of prescriptions) and prescription type (e.g. group treatment). To calculate the average coverage of veterinary practices from 2020 to 2023, the FSVO cross-referenced the list of all practices that received antibiotics from pharmaceutical companies in each year with the list of practices that prescribed antibiotics during the same period. Seasonality and autocorrelation in monthly prescription counts were evaluated using the Ljung–Box test applied to lags up to 12 months, capturing both short-term and annual autocorrelation patterns.^16^ Seasonal Extraction in ARIMA Time Series (SEATS) was created and analysed using the seasonal package (version 1.9).^17^ The Kendall’s tau correlation test was applied to determine the presence and direction of monotonic trends over time.^18^

### System architecture and data workflow

**Figure 1.**
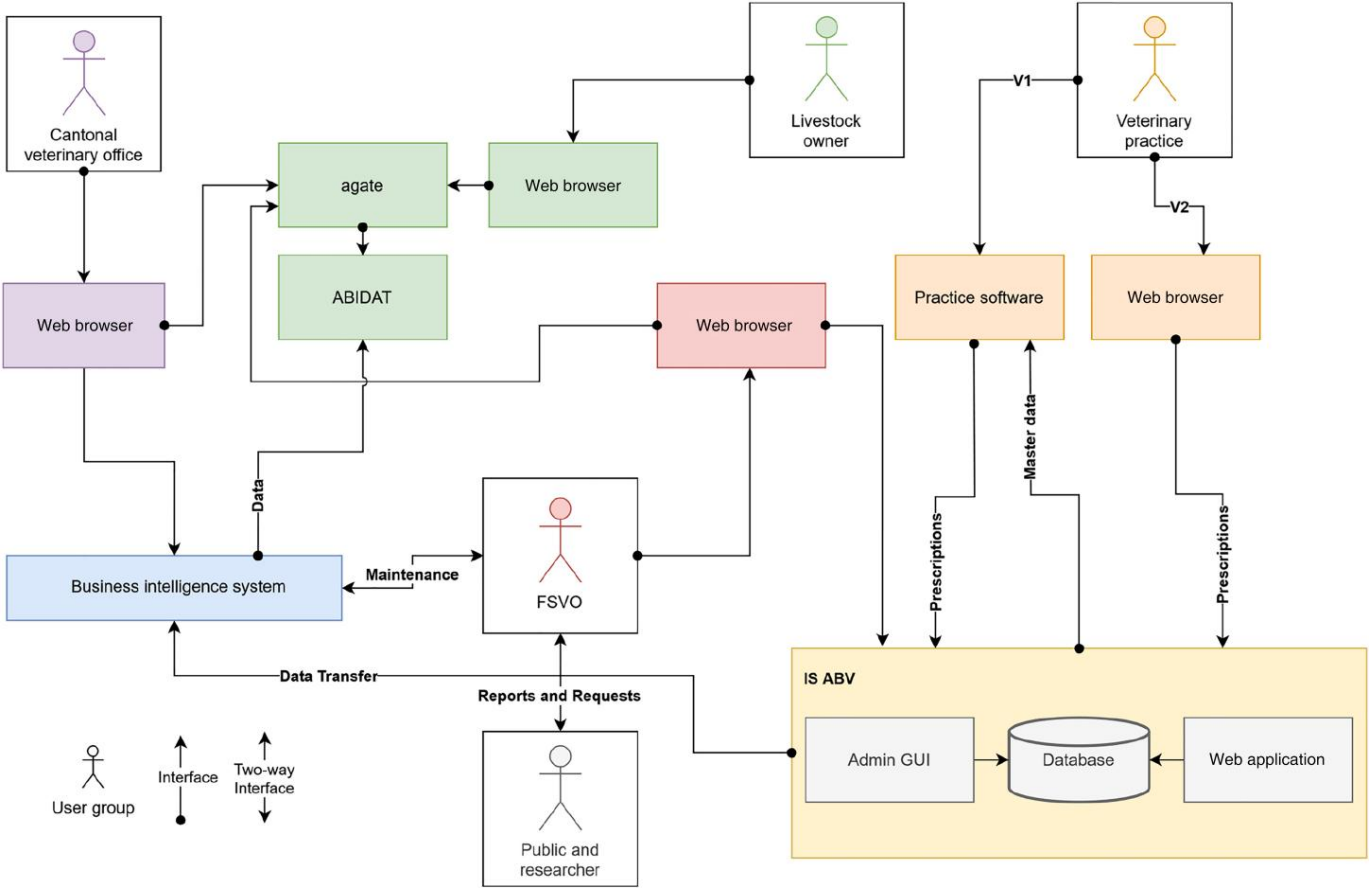
A simplified overview of the key components, main actors and interactions of the IS ABV architecture. agate, platform for agriculture applications; AMD, Animal Movement Database; GUI, graphical user interface.

All veterinarians in Switzerland must submit their antibiotic prescriptions electronically to the IS ABV server within one month.^9^ Veterinarians must register their practice with their Enterprise Identification Number (UID) in IS ABV. Veterinarians can then either enter their prescriptions via the web application or link their practice software via an interface to the IS ABV server (Figure 1). Although each prescription must be approved and sent to IS ABV individually, the practice software can use its metadata to pre-fill required fields, thus making the process semi-automatic and reducing the workload.

Owing to their complexity, certain prescription types can only be sent via the web application, such as oral group treatment. The prescription data are sent to the IS ABV server, and after being processed by the business logic layer (software architecture that is responsible for implementing the business logic of an application), they are stored in the IS ABV database (Figure 1). The prescription data is sent daily to the Extract, Transform, Load server using Secure File Transfer Protocol, converted there and then stored in the data warehouse, where it is accessible to Business Intelligence (BI) tools. The FSVO develops BI reports based on the processed data, which can then be used by the regional (cantonal) veterinary offices to assist with control measures in veterinary practices and livestock farms. In addition, the FSVO prepares annual reports for the public and processes the data to respond to public enquiries and for use in research projects. In addition, monthly basic individual reports with feedback are generated for each veterinary practice via the web application. These reports include all outliers and errors as well as a comprehensive list of all prescriptions received by the IS ABV server. Livestock owners can have access to a summary of their antibiotic prescriptions (i.e. how many and what type of antibiotics are being prescribed for their farms) through a portal of federal applications for livestock called ABIDAT (antibiotic data for farmers).

### Data collection and master data

Veterinarians can choose from three main prescription types, namely single treatment for either companion animals or farm animals and only for farm animals: group treatment (oral and non-oral) as well as dispensing on stock. Dispensing on stock is a specific practice in Switzerland whereby veterinarians may, under certain conditions, provide antibiotics to farmers with instructions for use, which the farmers may later then use for defined animal species and indications.^19^ The appendix contains definitions for each of the farm animals. For both single treatment types, veterinarians can register multiple animals that they treat individually. If the group size for orally treated farm animals is larger than the predefined size of 20 pigs, 50 poultry or rabbits and 10 of other species, the oral group treatment prescription type must be used. This must specifically be entered via the IS ABV web application because more information is needed to calculate the dose of substances per ration and animal. The amount of antibiotics is limited, and prophylactic use and critical antibiotics are prohibited in this prescription practice.

In all prescriptions, veterinarians must record information on the animal species, preparation, dose, frequency, duration and number of animals treated (full list in the Supplementary data). For companion animals and oral group treatments the weight of the treated animals is also recorded. For the other prescriptions, a reference weight for each animal species is predefined that can be changed by the veterinarian. Dispensing on stock prescriptions requires the least amount of information from veterinarians whereas oral group treatments requires the most. In addition to other regularly maintained master data, the FSVO provides lists of all preparations, active substances, animal species and production types online for download and via an interface for practice software.

### Data quality

The initial data analysis revealed that many of the prescriptions contained inaccuracies, such as incorrect dosages of antibiotics, incorrect treatment durations or incorrect numbers of animals treated. To ensure a more accurate analysis of antibiotic prescription data the FSVO applied a preparation-based median exclusion criterion. Specifically, the median dose per preparation was calculated for each production type, per animal (and per kilogram, where available), and per treatment day. Then, to provide an estimate of the maximum conceivable dosage for each preparation, the median was multiplied by 15. The factor of 15 was chosen based on expert opinion of the FSVO, which found that most major errors in IS ABV occurred above this threshold. However, this probably exceeds safe levels for many drugs and should not be considered a clinical maximum, but rather a threshold for identifying major errors. Prescriptions were classified as an error if the dosage administered exceeded the estimated maximum dosage per preparation.


$$\mathrm{Estimated}\;\mathrm{Maximum}\;\mathrm{Dosag}e_{\mathrm{Preparation}}=15\;\times \;\mathrm{Median}\left(\frac{\mathrm{Total}\;\mathrm{Dose}\;\mathrm{Prescribed}}{\mathrm{Number}\;\mathrm{of}\;\mathrm{Animals}\;\times \;\mathrm{Treament}\;\mathrm{Days}}\right)$$

In this study, the estimated annual error rate was calculated by dividing the number of prescriptions that exceeded the estimated maximum dosage per preparation by the total number of prescriptions for that year. To express this error rate per 1000 prescriptions, the value was multiplied by 1000:


$$\begin{array}{rl} & \mathrm{Estimated}\;\mathrm{Annual}\;\mathrm{Error}\;\mathrm{Rate}\;\mathrm{per}\;1000\\ & \;=\frac{\mathrm{Prescriptions}\;\mathrm{with}\;\mathrm{errors}\;\;}{\mathrm{Annual}\;\mathrm{prescriptions}\;\;}\times \;1000 \end{array}$$

It is important to note that prescriptions with errors can be corrected by the veterinarians themselves within two calendar years. Veterinarians can use the web application or practice software to deactivate an incorrect prescription and resend the corrected prescription.

## Results

### Practices

The annual comparison of the practices registered in IS ABV with practices buying antibiotics according to the distribution data (provided by the FSVO) showed an average coverage rate of 97% for the registrations since 2020.

In addition, after an initial catch-up period following the introduction of the system in 2020, we observed a decreasing trend from 2021 onwards in the number of practices prescribing antibiotics (Table 1). Between 2020 and 2023, the total number of practices prescribing exclusively for companion animals rose (+9.19%), while the number of practices prescribing solely for livestock decreased (−8.11%). Furthermore, the number of veterinary practices prescribing for both animal types decreased gradually over the same period, with a total decline of 6.54%.

**Table 1.** Evolution of the number of veterinary practices prescribing antibiotics for companion animals and livestock in Switzerland between 2020 and 2023

Year	Total	Companion animals (%)	Farm animals (%)	Mixed animals (%)
2020	1037	642 (61.91)	74 (7.14)	321 (30.95)
2021	1085	690 (63.59)	69 (6.36)	326 (30.05)
2022	1075	693 (64.47)	69 (6.42)	313 (29.12)
2023	1069	701 (65.58)	68 (6.36)	300 (28.06)

### Prescriptions

Most (96%) of the prescriptions were submitted via the practice software interface (v.1). The annual average number of prescriptions overall was 1 508 828 but had decreased by 1.36% since the start of the system (Table 2). Particularly prescriptions for dispensing on stock decreased by 24.27% from 218 154 (2020) to 165 199 (2023). Single treatments for companion animals increased to 636 847 in 2021 after the initial start and then decreased by 7.66% from 2021 to 2023.

**Table 2.** Number of antibiotic prescriptions per prescription types from 2020 to 2023 in Switzerland

Year	Prescriptions	Single treatment (companion)	Single treatment (farm)	Dispensing on stock (farm)	Group treatment (farm)
2020	1 484 307	600 936	655 138	218 154	10 079
2021	1 566 526	636 847	711 081	208 898	9 700
2022	1 520 377	609 463	715 992	185 854	9 068
2023	1 464 100	591 524	699 209	165 199	8 168

**Figure 2.**
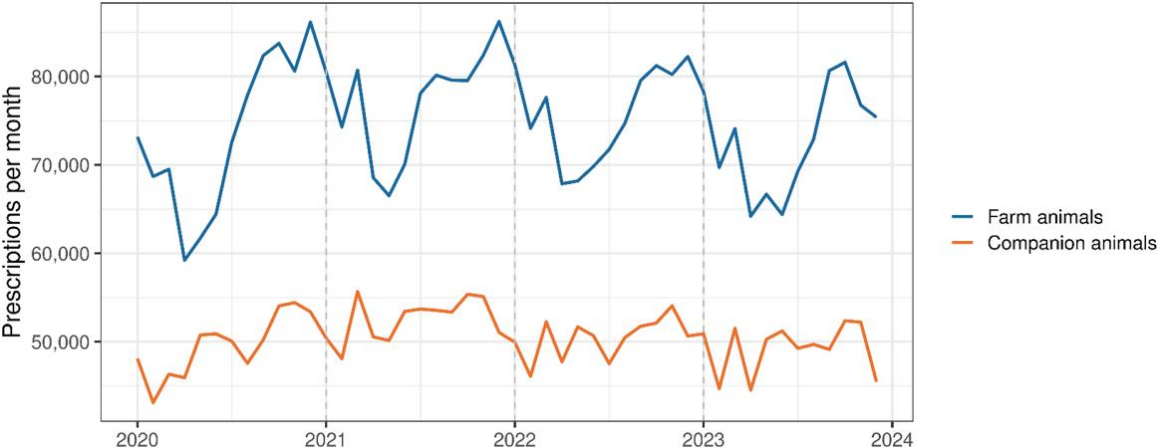
Monthly number of antibiotic prescriptions by type in Switzerland from January 2020 to December 2023.

**Figure 3.**
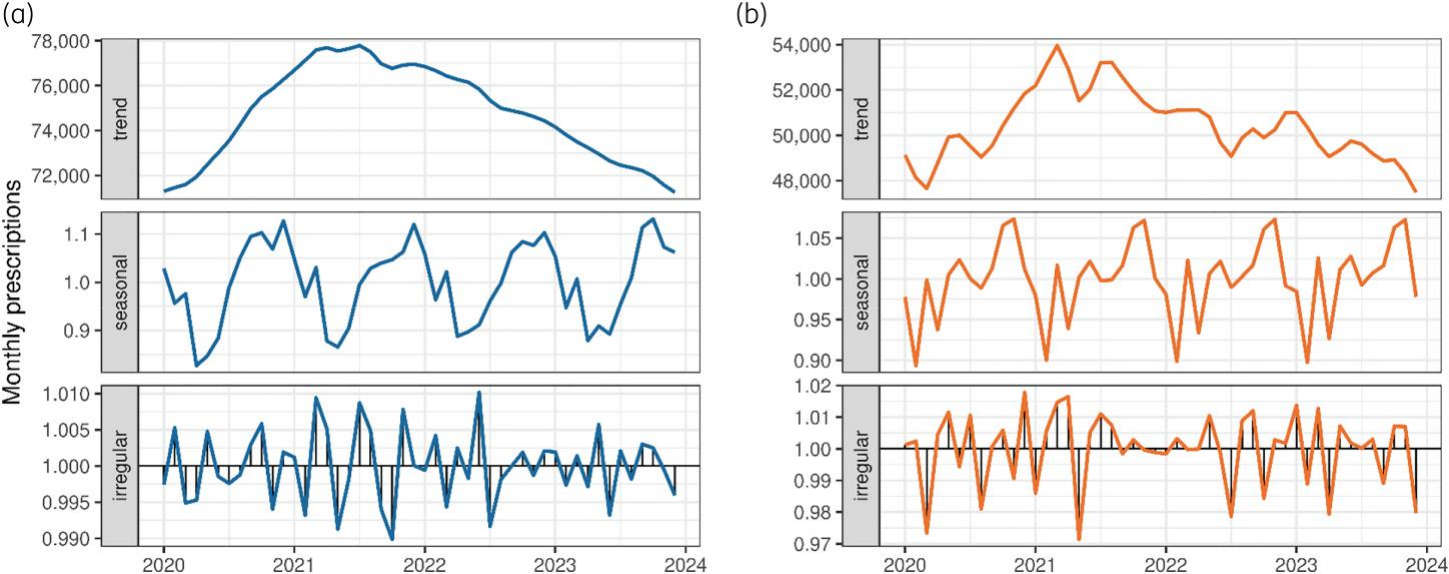
Decomposed time series of monthly prescriptions. (a) Prescriptions for farm animals, shown in blue and (b) prescriptions for companion animals, shown in orange. The trend shows the long-term direction of the data, the seasonal component captures regular patterns or cycles within the year and the irregular component represents the residuals or random noise after accounting for the trend and seasonal effects.

The monthly number of prescriptions for farm animals exhibited significant seasonality and autocorrelation, as indicated by the Ljung–Box test results (*χ*² = 117.769, degrees of freedom = 12, *P* value <0.001) (Figure 2). The number of prescriptions for farm animals increased in the early winter months. The seasonal trend in the number of prescriptions for companion animals was less pronounced (Figure 2), but similar variations were still observed in the decomposed time series. There was no significant autocorrelation for companion animals (*χ*² = 15.354, degrees of freedom = 12, *P* = 0.223) (Figure 3). However, when dogs and cats were separated, there was a seasonal pattern, with high monthly prescriptions for dogs in summer and the opposite high monthly prescriptions for cats in late autumn and early winter (Figure S1, available as Supplementary data at *JAC-AMR* Online). We also observed that February was the month with the lowest number of prescriptions prescribed for companion animals, and that there was a rapid increase in prescriptions in early spring. When adjusted for seasonality, there was evidence of a decreasing trend in prescriptions for both farm and companion animals. Kendall’s test showed a downward trend with a tau value of −0.27 (*P* = 0.007) for farm animals and a tau value of −0.227 (*P* = 0.023) for companion animals.

### Estimated annual error rate over time

**Figure 4.**
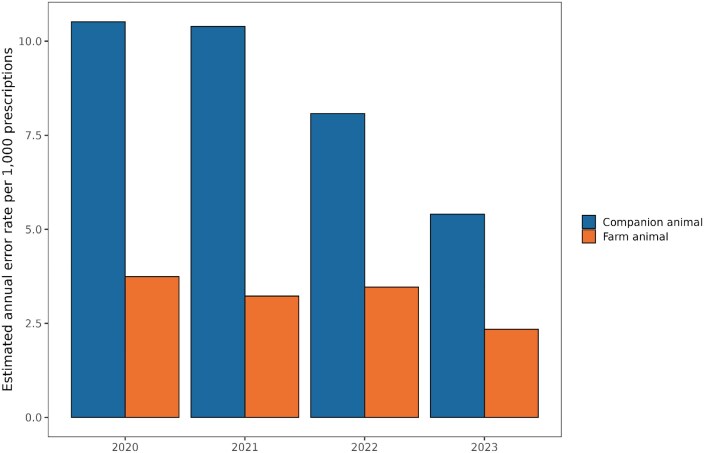
Evolution of the estimated annual error rate from 2020 to 2023 for companion and farm animals. The error rate indicates the proportion of prescriptions that exceed the estimated maximum dosage per preparation each year. This figure illustrates the approximate changes in prescription accuracy over time for both companion and farm animals.

The estimated number of errors has decreased since 2020 for both farm and companion animals (Figure 4). One exception was 2022, when farm animals experienced an increase in the estimated annual error rate. One of the most common errors found was confusion over the volume, weight and/or count units for each preparation required by the system when entering prescription data. Two examples of this are penicillin-containing veterinary medical products that often require millions of international units, and products sold as tablets and registered in IS ABV with the unit ‘pieces’ being confused with blisters or parts of cross-scored tablets.

## Discussion

### Practices

The comparison of practices registered in IS ABV and practices receiving antibiotics from pharmaceutical companies showed a high average coverage of 97% over the years, suggesting that the system effectively covers almost all relevant veterinary practices. The discrepancy was investigated and found to be mainly due to temporary changes in practices and business types. In Switzerland, the overall distribution of practices prescribing antibiotics has remained more or less stable between 2020 and 2023. We observed a slight decrease in the total number of practices prescribing antibiotics and a shift towards more practices prescribing only for companion animals. In 2023, fewer veterinary practices prescribed antibiotics exclusively for farm animals or for both companion and farm animals compared with previous years.

One reason for this is probably that there were fewer farms overall in 2023, so the number of veterinarians treating them should have declined.^20^ Economic and social changes in Switzerland, such as the ongoing decline in the number of small farms and pressure to reduce production costs, are reshaping the role of veterinary practices. Farm animal medicine must adapt to new conditions in line with the structural changes in agriculture. Cost pressures in agriculture are reducing the willingness of farmers to pay reasonable prices for veterinary services.^21^

Most Swiss veterinary practices are small and medium-sized enterprises with ∼10 veterinarians, but the trend is towards larger practices with more staff.^22^ Small animal practices are increasingly being sold to practice chains, where the business management can be centralized and the veterinarian can focus on medical services.^22^ This shift in practice structures highlights how broader organizational and economic changes are influencing both the professional roles of veterinarians.

### Prescriptions

For the relatively small veterinary landscape of Switzerland with an average of 1067 practices, we observed a national average of 125 736 prescriptions per month and an average of 1 508 828 prescriptions per year. Given the information contained in a prescription, this represented a substantial volume of data received. Interestingly, although the number of practices prescribing for companion animals has increased from 963 to 1001, the number of prescriptions for companion animals has decreased by 1.57%.

Initially we observed high levels of dispensing on stock prescriptions. This prescription type requires minimal data entry, making it the quickest, but should only be used for antibiotics dispensed on stock. The sharp decline in this category suggests that it is now being used more appropriately, possibly because it was misused during the initial launch of the system. The decline in use of this category has resulted from discussions with the Swiss Veterinary Association and the cantonal veterinary services, as well as knowledge exchange through newsletters, presentations to veterinary practices and other means of information sharing. Overall, we can say that there has been a significant decrease in prescriptions in farm and companion animals over the last 4 years, which is consistent with the decrease in national sales data from pharmaceutical companies.^6^

The increase in prescriptions for farm animals during the winter months was in line with general expectations, as animals are more susceptible to diseases such as respiratory infections when temperatures drop, and the stress of moving from outdoor grazing to indoor confinement can weaken their immune systems.^23^ Other major contributing factors include increased stocking density, as well as reduced ventilation and hygiene, all of which significantly heighten exposure to pathogens.^24^ In addition, farm animals in Switzerland are affected by seasonal changes, such as summer grazing and seasonal reproductive cycles. However, the number of prescriptions alone does not provide definitive evidence of higher treatment intensity of animals, which needs to be analysed in more detail using treatment intensity indicators such as the defined daily doses for animals, which was beyond the scope of this article. Compared with farm animal prescriptions, no overall trend was observed in companion animal prescriptions. Nevertheless, when we separated dogs and cats, we saw that the two showed opposite effects, levelling out the overall seasonality. Dogs had a higher prescription frequency in summer while cats had a higher prescription frequency in early winter. Hopman *et al.*^25^ investigated seasonal trends in AMU in companion animal clinics in the Netherlands and also found that dogs showed higher use in spring and summer compared with other companion animals.

### Estimated annual error rate over time

Since the introduction of IS ABV in 2020, the estimated annual error rate decreased, with one exception of 2022 for farm animals. It is important to note that it is only the prescriptions that were too high in dose are counted as errors. The estimated annual error rate was higher in companion animals than in farm animals. This may be because the entry of animal weights is mandatory in a companion animal prescription and may have introduced an additional opportunity for error. The cause of prescription errors is difficult to determine. Some veterinary practice software automatically calculates values within a prescription, such as the total drug amount based on doses per day and treatment duration. If the practice software generates incorrect values that go unnoticed by the veterinarian, or if incorrect information is entered manually, incorrect data may be submitted. In some systems, the total amount of antibiotics is calculated based on the selected preparation and treatment plan, so incorrect input could result in an inaccurate amount being reported to IS ABV. Such errors may be caused by staff training gaps, misleading software information or negligence on the part of the veterinarian. For the FSVO it was not feasible to clarify in each individual case whether the error was due to the veterinarians’ input, a transmission error or incorrect calculations. In the experience of the FSVO, misinterpretation of the unit to be reported in a prescription leads to most errors. A more advanced approach to tagging inaccuracies and list of the most common prescription errors is described in Schnidrig *et al.*^26^ In this pilot project, the aim was to develop a semi-automatic anomaly detection system using machine learning algorithms. In the long term, these tagged prescriptions will be used to provide additional feedback to veterinary practices for more appropriate error identification and correction.

Several measures have been taken by the FSVO to improve data quality. In early 2021, a monthly report was introduced to provide veterinary practices some basic feedback about their recorded prescriptions. Within this report, major discrepancies are flagged and reported to practices, allowing practices to regularly check the data received from the IS ABV server. An extended version of this report is made available to the cantonal veterinary services, to support routine inspections of veterinary pharmacies or farms. In addition, direct contact with the veterinarians helped reducing systematic data entry errors. In late 2023, the FSVO also introduced an online report (ABIDAT) for all farmers about the prescribed antibiotics. This allowed farmers to check which and how much antibiotics their veterinarian had prescribed for their farm and gave the possibility to report potential input errors. The decreasing trend in prescription errors could be explained by the measures taken to raise awareness among veterinary practices of the potential errors in the data collected, as well as increased familiarity with the system over time.

### Challenges and limitations

Implementing a nationwide data recording system such as IS ABV presents several practical and regulatory challenges that affect both the quality of the data and the functionality of the system. One of the key challenges was involving the practice software developers in the implementation of new changes, which has been one of the major issues of IS ABV. Under the current legislation, they are not obliged to participate directly. While developing and building IS ABV, the focus was on veterinarians and their involvement. At that time, the interface to the practice software was not a focus of attention. Sometimes, changes to the interface were not implemented within the specified timeframe, resulting in different data formats and ultimately a loss of information. Currently, there is no systematic way to verify that all prescriptions are entered by veterinarians. Cantonal veterinary offices, however, regularly inspect practices, reviewing medical supplies and prescriptions to detect discrepancies and promote compliance.

Since 2020, national data from IS ABV have been used to monitor antibiotic use. This has shown clear advantages over sales data from pharmaceutical companies. However, the time and resources required to enable data submission through practice software and improve initial data quality were underestimated. Prescription errors are likely to remain a challenge in the future. While the semi-automatic nature of IS ABV has solved some problems, it has also introduced new complexities, such as erroneous prescription presets, discrepancies between the practice software interface and the system’s requirements, and less attention to data quality when submitting data. In addition, the current estimate of the maximum conceivable dosage for each preparation is a static and more or less arbitrary construct. The advantages are that it provides a consistent upper limit for estimating maximum conceivable doses and allows standardized comparisons across years, helping the FSVO to identify systematic patterns and errors over time. Still a shift towards a more advanced approach as described in Schnidrig *et al.*^26^ would be preferable. The overall focus of the errors is on the veterinarians and the practice, as they are the ones entering the data, but it is equally important to recognize the shortcomings in communication between the FSVO and the practice software developers. Improvements are needed to ensure that critical updates and information are communicated clearly and shared effectively among the FSVO, cantonal veterinary offices, veterinarians and software developers.

### Strength and comparison with other systems

One of the major strengths of IS ABV is its access to more detailed data on prescriptions for farm and companion animals. Similar monitoring systems have been implemented in most European countries, and Sanders *et al.*^14^ provided a comprehensive overview and analysis of their key components. To date, IS ABV is the only system in Europe that systematically collects data on AMU in companion animals. This specificity provides valuable insights into AMU patterns in these animals, which are often under-represented in other systems. In addition, IS ABV's robust framework extends beyond large farms to include small farms, ensuring that AMU is accurately recorded in different agricultural settings. This inclusivity allows for a more complete understanding of AMU in different types of livestock production, contributing to better monitoring and management of antibiotic resistance.

### Outlook

The FSVO carries out national-level benchmarking, comparing antibiotic treatment patterns across different types of veterinary practice and animal category. Currently, meaningful benchmarks have been established for the treatment of pets (dogs, cats and horses) and poultry farms (all major categories). Benchmarking for cattle farms (rearing and fattening, dairy cows and other cows) is underway. Benchmarking of livestock veterinary practices will follow benchmarking of farms. This expansion will introduce sector-specific thresholds: action values that trigger interventions and signal values that serve as early warnings. Areas that are not suitable for benchmarking due to a small number of consultations or animal holdings will be identified, and alternative evaluations will be conducted. For instance, action and signal levels (thresholds for triggering intervention or monitoring) for laying hens have been discontinued as only a small percentage of farms use antibiotics. Planned system updates will require veterinarians to provide more detailed information on animal categories and the number of animals treated, particularly regarding dispensing to on stock.

In the future, IS ABV data will provide a comprehensive basis for benchmarking farms on their use of antibiotics against farms with the same production type. Benchmarking at both veterinary practice and farm level will be essential to identify areas for improvement and promote best practice. The development of a dashboard to track AMU, coupled with the availability of real-time data, will allow for continuous evaluation and improvement of antibiotic stewardship across the industry. The objectives of AMU data collection are complex and interdependent. They can be divided into the monitoring of consumption, the identification of areas with high antibiotic consumption, and benchmarking to identify and address high consumers in animal husbandry and veterinary practices. For all three objectives, however, data on antibiotic consumption is only a part of the necessary data; good data on animal populations at a resolution that allows comparisons is also needed.

### Conclusion

Since 2020, the national information system of antibiotic usage (IS ABV) in Switzerland has been successfully implemented and has shown continuous improvement in data quality. After adjusting for seasonality, we have observed a decline in antibiotic prescriptions for companion and farm animals which is a strong indicator of a meaningful change in prescribing behaviour. In addition, the estimated annual error rate in prescriptions has steadily decreased since the system’s implementation, indicating that veterinarians are becoming more accurate in reporting their AMU. Benchmarking at both the practice and farm levels, along with continuous monitoring of AMU, will be key to identifying improvement areas and promoting best practices. Overall, IS ABV will enhance efforts to fight antibiotic resistance, track usage across various sectors and support authorities in making informed decisions for future actions.

## Supplementary Material

dlaf199_Supplementary_Data

## Supplementary data

Figure S1 is available as Supplementary data at *JAC-AMR* Online.
