# Supplementary material for: Architecture, goals and challenges of the Swiss Information System for Antibiotics in Veterinary Medicine (IS ABV)
Source: JAC Antimicrob Resist. 2025 Oct 27;7(5):dlaf199. doi: 10.1093/jacamr/dlaf199 (PMC12557320; doi:10.1093/jacamr/dlaf199)
Supplement: dlaf199_Supplementary_Data [file dlaf199_supplementary_data.docx]

# Supplementary material

## Farm animals

Farm animals are defined as animals of species that may be used for food production in accordance with food legislation or for feed production in accordance with animal disease legislation, and bees.

## Table for species

Supplementary table 1: All species that can be prescribed within IS ABV.

| **Category** | **Genus** | **Type of use** |
| --- | --- | --- |
| Companion animal | Companion animal | Companion animal |
| Companion animal | Equid | Equid |
| Companion animal | Cat | Cat |
| Companion animal | Dog | Dog |
| Companion animal | Other | Other |
| Farm animal | Farm animal | Farm animal |
| Farm animal | Cattle | Cattle |
| Farm animal | Cattle | Rearing calf |
| Farm animal | Camelids | Camelids |
| Farm animal | Farmed game | Farmed game |
| Farm animal | Cattle | Veal calf |
| Farm animal | Cattle | Rearing cattle |
| Farm animal | Cattle | Beef cattle |
| Farm animal | Cattle | Dairy cow |
| Farm animal | Cattle | Suckler cow |
| Farm animal | Cattle | Suckler calf |
| Farm animal | Pig | Pig |
| Farm animal | Pig | Suckling piglet / suckler |
| Farm animal | Pig | Weaned piglet / weaner |
| Farm animal | Pig | Fattening pig |
| Farm animal | Pig | Gilt |
| Farm animal | Pig | Lactating sow |
| Farm animal | Pig | Boar |
| Farm animal | Pig | Non-lactating sow |
| Farm animal | Poultry | Poultry |
| Farm animal | Poultry | Laying hens (rearing) |
| Farm animal | Poultry | Laying hens (laying phase) |
| Farm animal | Poultry | Broiler chicken |
| Farm animal | Poultry | Broiler turkey |
| Farm animal | Poultry | Broiler breeder (laying phase) |
| Farm animal | Poultry | Layer breeder (rearing) |
| Farm animal | Poultry | Layer breeder (laying phase) |
| Farm animal | Poultry | Other (poultry) |
| Farm animal | Poultry | Broiler breeder (rearing phase) |
| Farm animal | Sheep | Sheep |
| Farm animal | Sheep | Sheep, young animal |
| Farm animal | Sheep | Sheep, adult |
| Farm animal | Goat | Goat |
| Farm animal | Goat | Goat, young animal |
| Farm animal | Goat | Goat, adult |
| Farm animal | Rabbit | Rabbit |
| Farm animal | Rabbit | Rabbit, adult |
| Farm animal | Rabbit | Rabbit, young animal |
| Farm animal | Fish | Fish |
| Farm animal | Camelids | Camelids |
| Farm animal | Farmed game | Farmed game |
| Laboratory and zoo animals | Laboratory and zoo animals | Laboratory and zoo animals |
| Laboratory and zoo animals | Laboratory animal | Laboratory animal |
| Laboratory and zoo animals | Laboratory animal | Rabbit |
| Laboratory and zoo animals | Laboratory animal | Guinea pig |
| Laboratory and zoo animals | Laboratory animal | Mouse |
| Laboratory and zoo animals | Laboratory animal | Rat |
| Laboratory and zoo animals | Laboratory animal | Other mammal |
| Laboratory and zoo animals | Laboratory animal | Bird |
| Laboratory and zoo animals | Laboratory animal | Reptile |
| Laboratory and zoo animals | Laboratory animal | Other laboratory animal |
| Zoo animal | Zoo animal | Zoo animal |
| Zoo animal | Zoo animal | Mammal |
| Zoo animal | Zoo animal | Bird |
| Zoo animal | Zoo animal | Reptile |
| Zoo animal | Zoo animal | Fish |
| Zoo animal | Zoo animal | Other zoo animal |

Table of all required input fields

Supplementary table 2: All the necessary inputs for an IS ABV prescription. The specific fields to be completed may vary depending on the type of prescription, with additional or fewer fields needed accordingly. The UID is the government-issued identification number for companies (here veterinary practices). The AMD identifier (government-issued) is the unique identifier assigned to each farm as an epidemiological unit for animal movement tracing in Switzerland. More optional inputs can be found on the FSVO website.

| **Name** | **Short description** | **Format** |
| --- | --- | --- |
| dateOfVisit | Date of visit | dateTime |
| recipeNumber | Unique prescription number | string |
| veterinaryPracticeUID | Practice registration number | string |
| veterinaryPracticeAddress | Veterinary practice address | string |
| Usage | Animal used for food or non-food purposes. | string |
| species | Species treated | string |
| amdIdentifer | AMD number of the farm holding the animals | string |
| address | Adress of the farm | string |
| treatingAnimalsNumber | How many animals were treated | positiveInteger |
| animalWeight | Animal weight (mandatory for group treatments and companion animals) | double |
| diagnosis | Diagnosis based on predefined list | string |
| organSystem | Organ system based on predefined list | string |
| preparationID | ID of the preparation | string |
| dailyDose:animalDosis | Dose per animal | double |
| dailyDose:treatmentDays | Days the treatment lasted | positiveInteger |
| dailyDose:servingMode | Frequency of treatment | string |
| preparation:givenAmount | Given amount of the preparation | double |
| criticalSubstanceConfirmed | Is the active substance on the list of critical substances | Boolean |

## Time series for dogs and cats

The Box-Ljung test conducted on monthly dog prescriptions yielded a chi-squared value of 30.40205, with 12 degrees of freedom and a p-value of 0.00242829.


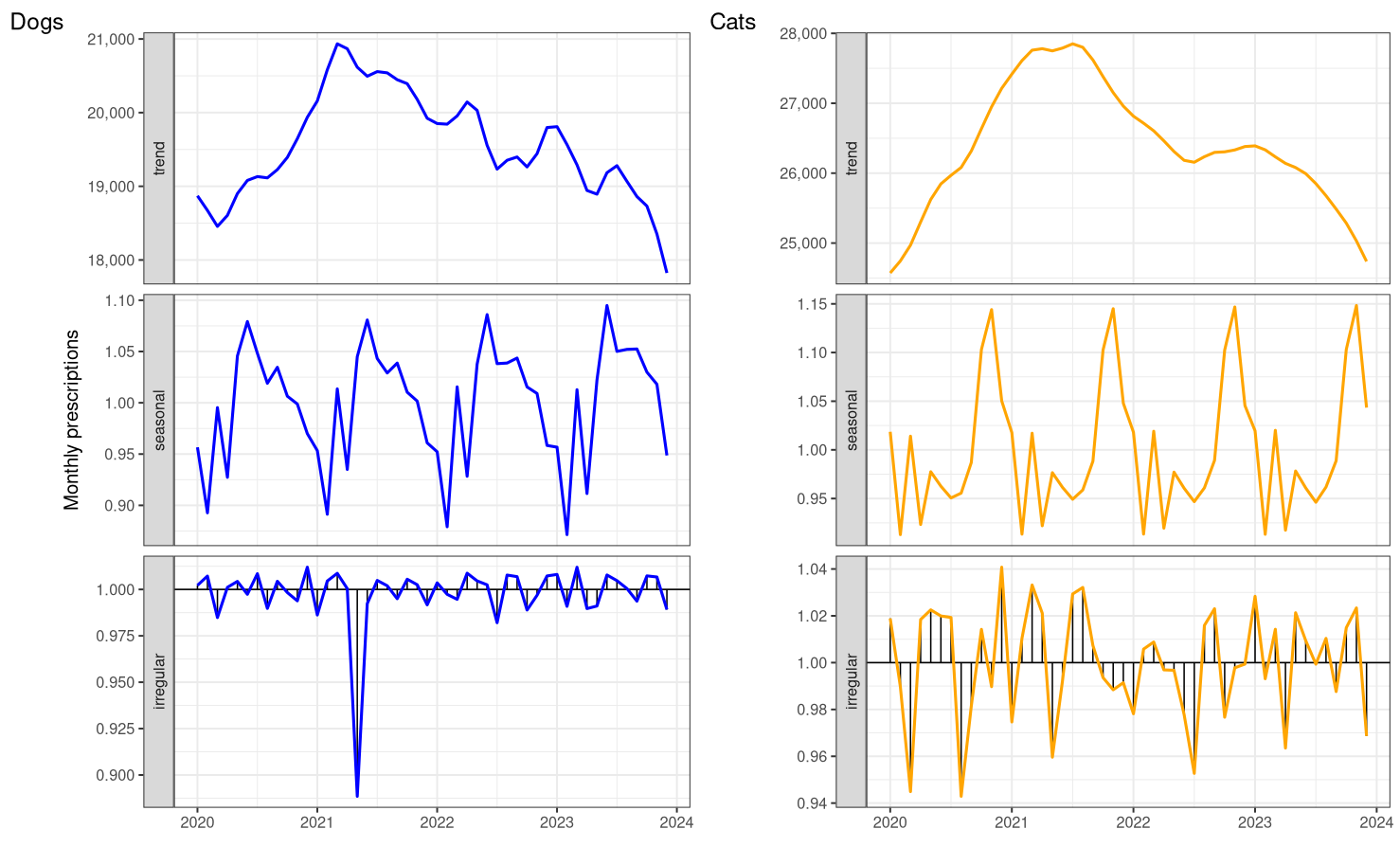
The Box-Ljung test conducted on monthly cat prescriptions yielded a chi-squared value of 41.0702, with 12 degrees of freedom and a p-value of 0.0000476959.

Supplementary Figure 1: Decomposed time series of monthly prescriptions. Panel (A) on the left, shown in blue, represents prescriptions for dogs, while panel (B) on the right, shown in orange, represents prescriptions for cats.
